# Prevalence of low central venous oxygen saturation in the first hours of intensive care unit admission and associated mortality in septic shock patients: a prospective multicentre study

**DOI:** 10.1186/s13054-014-0609-7

**Published:** 2014-11-06

**Authors:** Thierry Boulain, Denis Garot, Philippe Vignon, Jean-Baptiste Lascarrou, Arnaud Desachy, Vlad Botoc, Arnaud Follin, Jean-Pierre Frat, Frédéric Bellec, Jean-Pierre Quenot, Armelle Mathonnet, Pierre-François Dequin, for the Clinical Research in Intensive Care and Sepsis (CRICS) Group

**Affiliations:** Medical-Surgical Intensive Care Unit, Regional Hospital Center, Orléans, France; Medical Intensive Care Unit, University Hospital, Tours, France; Medical-Surgical Intensive Care Unit, University Hospital, Limoges, France; CIC-P 1435, Inserm U1092, Limoges, France; Medical-Surgical Intensive Care Unit, District Hospital Center, La Roche-sur-Yon, France; Medical-Surgical Intensive Care Unit, District Hospital Center, Angoulême, France; Medical-Surgical Intensive Care Unit, District Hospital Center, Saint-Malo, France; Medical-Surgical Intensive Care Unit, District Hospital Center, Argenteuil, France; Medical Intensive Care Unit, University Hospital, Poitiers, France; Medical-Surgical Intensive Care Unit, District Hospital Center, Montauban, France; Medical Intensive Care Unit, University Hospital, Dijon, France; INSERM, U866, Dijon, France; Clinical Research in Intensive Care and Sepsis (CRICS) Group, Tours, France

## Abstract

**Introduction:**

In septic shock patients, the prevalence of low (<70%) central venous oxygen saturation (ScvO2) on admission to the intensive care unit (ICU) and its relationship to outcome are unknown. The objectives of the present study were to estimate the prevalence of low ScvO2 in the first hours of ICU admission and to assess its potential association with mortality in patients with severe sepsis or septic shock.

**Methods:**

This was a prospective, multicentre, observational study conducted over a one-year period in ten French ICUs. Clinicians were asked to include patients with severe sepsis or septic shock preferably within 6 hours of ICU admission and as soon as possible without changing routine practice. ScvO2 was measured at inclusion and 6 hours later (H6), by blood sampling.

**Results:**

We included 363 patients. Initial ScvO2 below 70% was present in 111 patients and the pooled estimate for its prevalence was 27% (95% Confidence interval (95%CI): 18% to 37%). At time of inclusion, among 166 patients with normal lactate concentration (≤2 mmol/L), 55 (33%) had a low initial ScvO2 (<70%), and among 136 patients who had already reached the classic clinical endpoints for mean arterial pressure (≥65 mmHg), central venous pressure (≥8 mmHg), and urine output (≥0.5 mL/Kg of body weight), 43 (32%) had a low initial ScvO2 (<70%). Among them, 49% had lactate below 2 mmol/L. The day-28 mortality was higher in case of low initial ScvO2 (37.8% versus 27.4%; *P* = 0.049). When adjusted for confounders including the Simplified Acute Physiology Score and initial lactate concentration, a low initial ScvO2 (Odds ratio (OR) = 3.60, 95%CI: 1.76 to 7.36; *P* = 0.0004) and a low ScvO2 at H6 (OR = 2.18, 95%CI: 1.12 to 4.26; *P* = 0.022) were associated with day-28 mortality by logistic regression.

**Conclusions:**

Low ScvO2 was common in the first hours of admission to the ICU for severe sepsis or septic shock even when clinical resuscitation endpoints were achieved and even when arterial lactate was normal. A ScvO2 below 70% in the first hours of ICU admission and six hours later was associated with day-28 mortality.

**Electronic supplementary material:**

The online version of this article (doi:10.1186/s13054-014-0609-7) contains supplementary material, which is available to authorized users.

## Introduction

Central venous oxygen saturation (S_cv_O_2_) has long been studied as a prognostic marker and resuscitation end-point in patients with shock [1]. It is an imperfect surrogate of mixed venous oxygen saturation (S_v_O_2_) because it reflects the oxygen supply-to-consumption ratio of the upper part of the body only. However, S_cv_O_2_ is simple to measure either continuously or intermittently, and spontaneous or therapy-induced changes in S_cv_O_2_ and S_v_O_2_ are closely correlated [2].

Based on these principles, Rivers *et al*. [3] showed that an early therapeutic strategy that includes aiming for the rapid normalization of S_cv_O_2_ (≥70%) in patients suffering from severe sepsis or septic shock at presentation to the emergency department could improve survival. Since then, international guidelines have recommended targeting S_cv_O_2_ at ≥70% during the first 6 hours of care in patients presenting with severe sepsis or septic shock [4]. However, this recommendation remains controversial. Indeed, resuscitation protocols including an S_cv_O_2_ target have been mostly tested in the emergency department [5-7], not after ICU admission. Importantly, after ICU admission, targeting normal S_cv_O_2_ is deemed to have little place, as S_cv_O_2_ is frequently considered to be already normalized on ICU admission [8]. However, the prevalence of low S_cv_O_2_ (<70%) in ICU septic patients is poorly known. It has been assessed only in two prospective studies of limited size [9,10], while other studies have only reported the mean initial value of S_cv_O_2_ [8,11]. In addition, the relationship between low S_cv_O_2_ and outcome is still unknown.

Since the Rivers study [3], the idea that the relationship between low S_cv_O_2_ and fatal outcome in septic patients has been widely demonstrated has remained. Up to now, however, such a relationship has not been demonstrated in multicentre prospective studies performed either in the emergency department [12] or in the ICU [10]. Accordingly, the aim of the present multicentre observational prospective study was to estimate the prevalence of low S_cv_O_2_ in patients presenting with severe sepsis or septic shock after ICU admission, and to assess its potential association with outcome.

## Materials and methods

The study took place in 10 French medical-surgical adult ICUs (4 university hospitals, 1 regional and teaching hospital, and 5 general hospitals) with a combined total of 154 ICU beds. Participating ICUs were asked to screen consecutive patients presenting with sepsis and circulatory failure for potential inclusion in the study, over a period left at their discretion, from July 2011 to June 2012.

Clinicians were asked to include patients with severe sepsis or septic shock, preferably within 6 hours of ICU admission, and as soon as possible without changing routine practice. Patients were included if they had circulatory failure of septic origin (that is, severe sepsis or septic shock [13]) within 12 hours after ICU admission, and had an intra-arterial and superior vena cava (internal jugular or subclavian) catheter in situ. Circulatory failure was defined by either the use of vasopressor (septic shock) or, in case of severe sepsis, by a mean arterial pressure <65 mmHg or a systolic arterial pressure <90 mmHg at least twice over a 15-minute period, associated with at least one condition reflecting tissue hypoperfusion or low flow state (see Table 1, Criteria for circulatory failure at inclusion). Patients were not included in the case of brain death, admission following cardiac arrest, or if imminent death was expected.

**Table 1:** **Demographics, clinical characteristics, treatment and outcome in 363 patients**

**Variable**	**Value**
**Male sex**	231 (63.6)
**Age, years**	65.8 ± 14.1
**Severe sepsis at inclusion**	25 (6.9)
**Septic shock at inclusion**	338 (93.1)
**Nosocomial infection**	98 (27.0)
**Source of infection**	
Lung-pleura	165 (45.7)
Abdomen	83 (23.0)
Urine	45 (12.5)
Skin, bones, joints	30 (8.3)
Catheter	7 (1.9)
Central nervous system	6 (1.7)
Endocarditis	6 (1.7)
Other	8 (2.2)
Source not identified	11 (3.0)
**Underlying disease**	
Chronic obstructive pulmonary disease	63 (17.4)
Arterial hypertension	198 (54.5)
Chronic heart failure	36 (9.9)
Liver cirrhosis	27 (7.4)
Chronic haemodialysis	8 (2.2)
Type 1 diabetes	15 (4.1)
Type 2 diabetes	85 (23.4)
Immunocompromized state	110 (30.3)
Active solid cancer	45 (12.4)
Active lymphoma or leukaemia	22 (6.1)
Myeloproliferative syndrome	12 (3.3)
Recent (<6 months) chemotherapy or radiotherapy	42 (11.6)
Neutropenia (absolute neutrophil count <1,000/mm^3^)	27 (7.4)
HIV seropositivity	4 (1.1)
Solid organ or bone marrow transplantation	6 (1.7)
Steroids therapy	41 (11.3)
Other immunosuppressive therapy	14 (3.9)
**Criteria for circulatory failure at inclusion (in addition to recent or ongoing hypotension)**	
Tachycardia (heart rate >110 beats/minute)	195 (53.7)
Urine output <0.5 mL/hour per Kg of body weight	147 (40.5)
Capillary refill time >2 seconds	48 (13.2)
Cyanosis in the absence of severe hypoxemia	56 (15.4)
Skin mottling	167 (46.0)
Altered consciousness	77 (21.2)
Arterial lactate >2 mmol/L on admission	190 (52.3)
Epinephrine or norepinephrine administration	338 (93.1)
**Therapy**	
Invasive mechanical ventilation on admission	128 (35.2)
Invasive mechanical ventilation during ICU stay	305 (84.0)
Norepinephrine on admission	136 (37.5)
Epinephrine on admission	10 (2.8)
Norepinephrine or epinephrine during ICU stay	356 (98.1)
Dobutamine on admission	12 (3.3)
Transfusion of blood products between zero hours and 24 hours	58 (16.6)
Renal replacement therapy during ICU stay	84 (23.1)
**Initial arterial blood lactate concentration, mmol/L** ^**a**^	3.2 ± 3.0
	median 2.2 (IQR 0.95 to 4.45)
**McCabe classification** [14]	
0	223 (61.4)
1	96 (26.4)
2	44 (12.1)
**Simplified acute physiology score II** [15]	56.8 ± 20.0
**SOFA score** [16] **(highest value from H0 to H24)**	10.3 ± 3.4
**ICU death**	102 (28.1)
**Day-28 death**	111 (30.6)

Categorical variables are expressed as absolute counts (%) and continuous variables as mean ± SD unless otherwise specified. ^a^Initial arterial blood lactate concentration was available in 358/363 patients (not measured in 5 patients because of technical problem). SOFA, Sequential organ failure assessment.

### Measurements and data collection

The measurement of S_cv_O_2_ was performed as soon as possible (at the time defined as zero hours (H0)) by sampling blood from the superior vena cava through the central venous catheter, and at 6 hours after inclusion (H6). S_cv_O_2_ was either calculated from blood gas analysis by a standard blood gas analyser in four centres, or measured by a co-oximeter in six centres. Concomitantly, arterial blood was drawn at each time point for blood gas analysis and lactate measurement. Other demographic, clinical and laboratory data prospectively recorded are provided in Additional file 1. Independent data monitoring was conducted in each site to ascertain the accuracy of recorded information.

The Ethics Committee of the teaching hospital of Limoges, France, approved the protocol for all hospitals involved (agreement number 65-2011-11) and waived the need for prior informed consent because the study procedures fulfilled the criteria of a non-interventional study as defined by the French Law [17]. Patients’ next-of-kin and then patients themselves if they regained capacity were informed of their participation and their right to refuse the use of the obtained data was clearly established.

Haemodynamic monitoring and treatment of shock followed international [18] and national guidelines [19] that corresponded to standard of care of the participating centres. However, none of the participating ICUs had implemented systematic treatment algorithms based on S_cv_O_2_ monitoring.

### Study objective

The study primary objective was to estimate the prevalence of low S_cv_O_2_ (<70%, but other thresholds were also tested) and to assess the possible association between low S_cv_O_2_ and day-28 mortality.

### Data reporting and statistical analysis

Categorical data are expressed as percentages, and continuous variables are expressed as means ± SD or medians and IQR, as appropriate. The prevalence and 95% CI of S_cv_O_2_ < 70% was estimated taking into account participating centres as a random effect [20]. The S_cv_O_2_ < 70% and other thresholds of initial S_cv_O_2_ values determined by locally weighted scatterplot smoothing (LOWESS) [21] were examined for their potential association with day-28 mortality.

Each given threshold of the initial S_cv_O_2_ value was entered in a mixed-effect logistic regression model, with participating centres entered as a random effect, and adjusted for all covariables available at H0 and linked to day-28 mortality with a *P*-value <0.15, by univariate analysis. The covariables were eliminated using the backward method until the final logistic model with the best fit (as assessed by the Akaike information criterion [15]) was reached. Based on the results of a retrospective study performed in one participating ICU [22], we calculated that 1-year study duration would allow the inclusion of at least 350 patients with 100 fatalities, which would allow multivariate evaluation of ten covariables, including S_cv_O_2_.

We re-ran the logistic analyses in predefined subgroups of patients (for example, mechanical ventilation or not at H0, patients below or above median values of continuous variables such as initial lactate level or amount of volume expansion before inclusion, et cetera).

Percentages were compared using the Fisher exact test, chi-square test, or chi-square test for trend (Cochran-Armitage test), and continuous variables were compared using the unpaired Student *t*-test or by analysis of variance when appropriate. Unadjusted comparisons of survival curves were performed using the log-rank test. Adjusted odds ratios (OR) are given with the 95% CI. A two-tailed *P*-value <0.05 was considered statistically significant. Mixed-effect logistic regression was performed using the lme4 package of R 2.15.2 [23].

## Results

**Figure 1:**
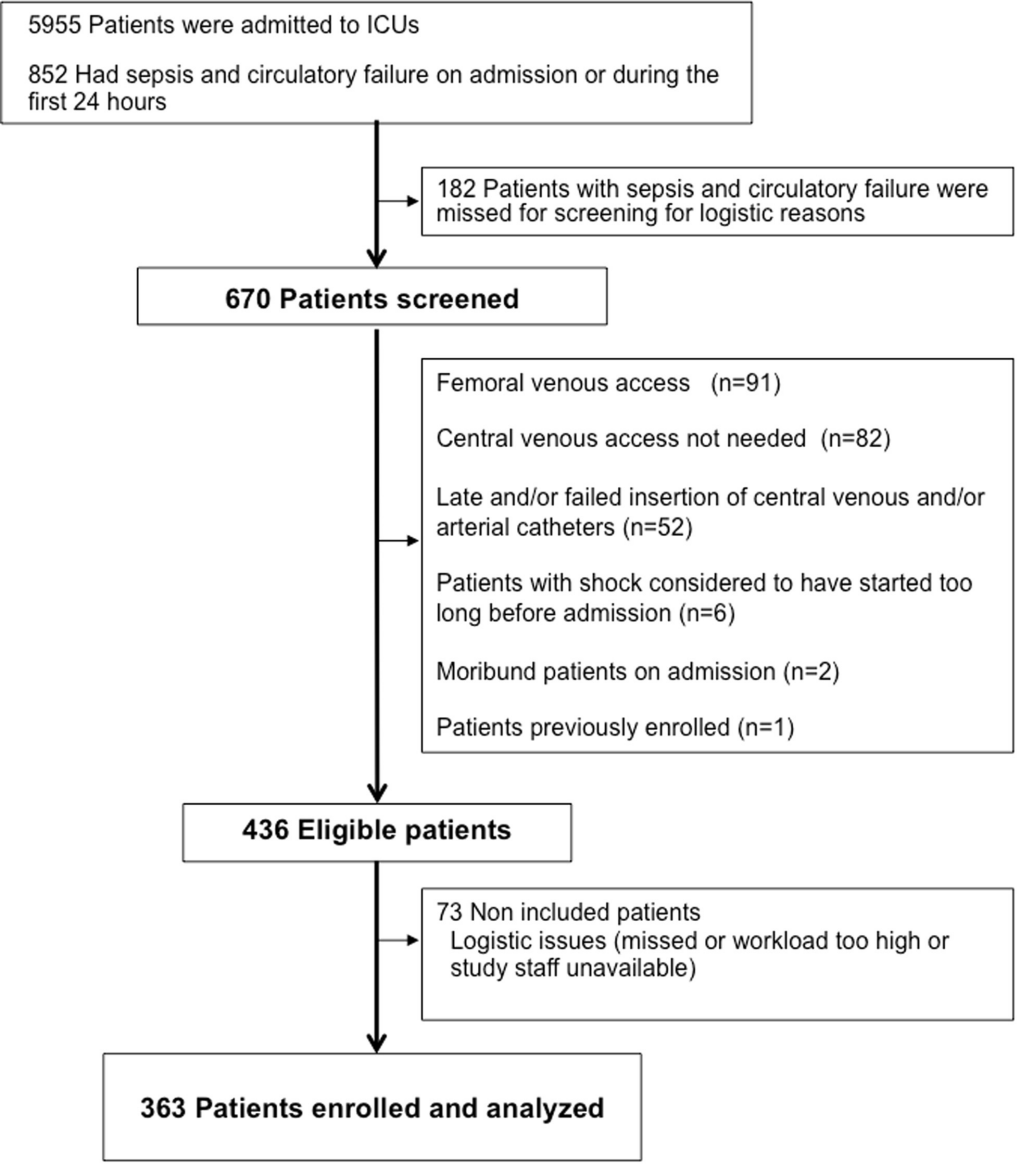
**Flow diagram.**

We screened 670 patients with severe sepsis or septic shock. Among them, 363 patients were enrolled (Figure 1). The Simplified acute physiology score (SAPSII) [24] and ICU death rates were similar in enrolled and non enrolled patients (see Additional file 2).

Demographic and clinical characteristics are shown in Table 1. The median time between ICU admission and inclusion (H0), and between the time at which criteria for inclusion were satisfied and inclusion, was 211 minutes (IQR 83 to 339) and 189 minutes (IQR 84 to 292), respectively. The median time between severe sepsis identification and inclusion (H0) was 7.6 hours (IQR 2 to 13.2). Details of resuscitation administered before inclusion and time of inclusion in patients classified according to their origin (transferred from another hospital, from the ward, or from the emergency department) are shown in Table 2.

**Table 2:** **Time of inclusion and resuscitation administered before inclusion**

	**Patients transferred from another hospital**	**Patients transferred from the ward**	**Patients transferred from the emergency department**
	**(n = 93)**	**(n = 95)**	**(n = 175)**
Patients intubated before inclusion	73 (78%)	72 (77%)	118 (67%)
Amount of fluid administered before inclusion, mL/Kg of body weight			
	31	35	36
	(12 to 50)	(19 to 51)	(20 to 52)
Patients treated with vasopressors before inclusion (epinephrine or norepinephrine)			
	84 (88%)	82 (90%)	155 (89%)
Dosage of vasopressors (epinephrine + norepinephrine), μg/Kg/min			
	0.3	0.26	0.40
	(0.09 to 0.51)	(0.12 to 0.38)	(0.13 to 0.66)
Patients treated with dobutamine before inclusion			
	3 (3%)	3 (3%)	10 (6%)
Time between severe sepsis identification and inclusion, minutes			
	482	396	468
	(220 to 510)	(40 to 847)	(188 to 696)
Time between ICU admission and inclusion, minutes			
	185	246	206
	(53 to 317)	(92 to 400)	(97 to 315)
Time between satisfaction of inclusion criteria and inclusion, minutes			
	231	161	185
	(110 to 352)	(60 to 262)	(93 to 277)

Continuous variables are expressed as median and IQR, and categorical variables as number (%).

The mean initial S_cv_O_2_ value was 74.1 ± 11.0% and was not different between survivors and non survivors at day 28 (74.6 ± 10.0% versus 73.0 ± 12.9%, respectively; *P* = 0.21).

### Prevalence of low initial S_cv_O_2_

Initial S_cv_O_2_ < 70% was present in 111 patients and the pooled estimate for its prevalence, taking into account participating centres as a random effect, was 27% (95% CI 18%, 37%). At H0, 136 patients (37%) had reached the classic clinical endpoints for mean arterial pressure (≥65 mmHg), central venous pressure (≥8 mmHg), and urine output (≥0.5 mL/Kg of body weight). Among them, 32% (43/136) had an initial S_cv_O_2_ < 70%, with normal lactate concentration (≤2 mmol/L) in 21 (49%) patients. At H0, 166 patients (46%) had normal lactate concentration (≤2 mmol/L). Among them, 55 (33%) had an initial S_cv_O_2_ < 70%. The delay between severe sepsis identification and inclusion and the amount of fluids administered for resuscitation before inclusion had no influence on the mean initial S_cv_O_2_ values or on the proportions of patients with initial S_cv_O_2_ < 70% (see Additional file 3).

### Crude day-28 death rate and initial S_cv_O_2_ level

As determined by visual inspection of the LOWESS plot (see Additional file 4), we assessed the association between day-28 mortality and initial S_cv_O_2_ < 70%, <75%, and >85%. The day-28 death rate was significantly higher in patients with initial S_cv_O_2_ < 70% than in patients with initial S_cv_O_2_ ≥ 70% (42/111 (37.8%) versus 69/252 (27.4%), respectively; *P* = 0.049), whereas it was similar in patients with initial S_cv_O_2_ below or above the other thresholds examined (all *P*-values >0.35) (see Additional file 5: Table E8).

### Association between S_cv_O_2_ and mortality when adjusted for potential confounders

**Figure 2:**
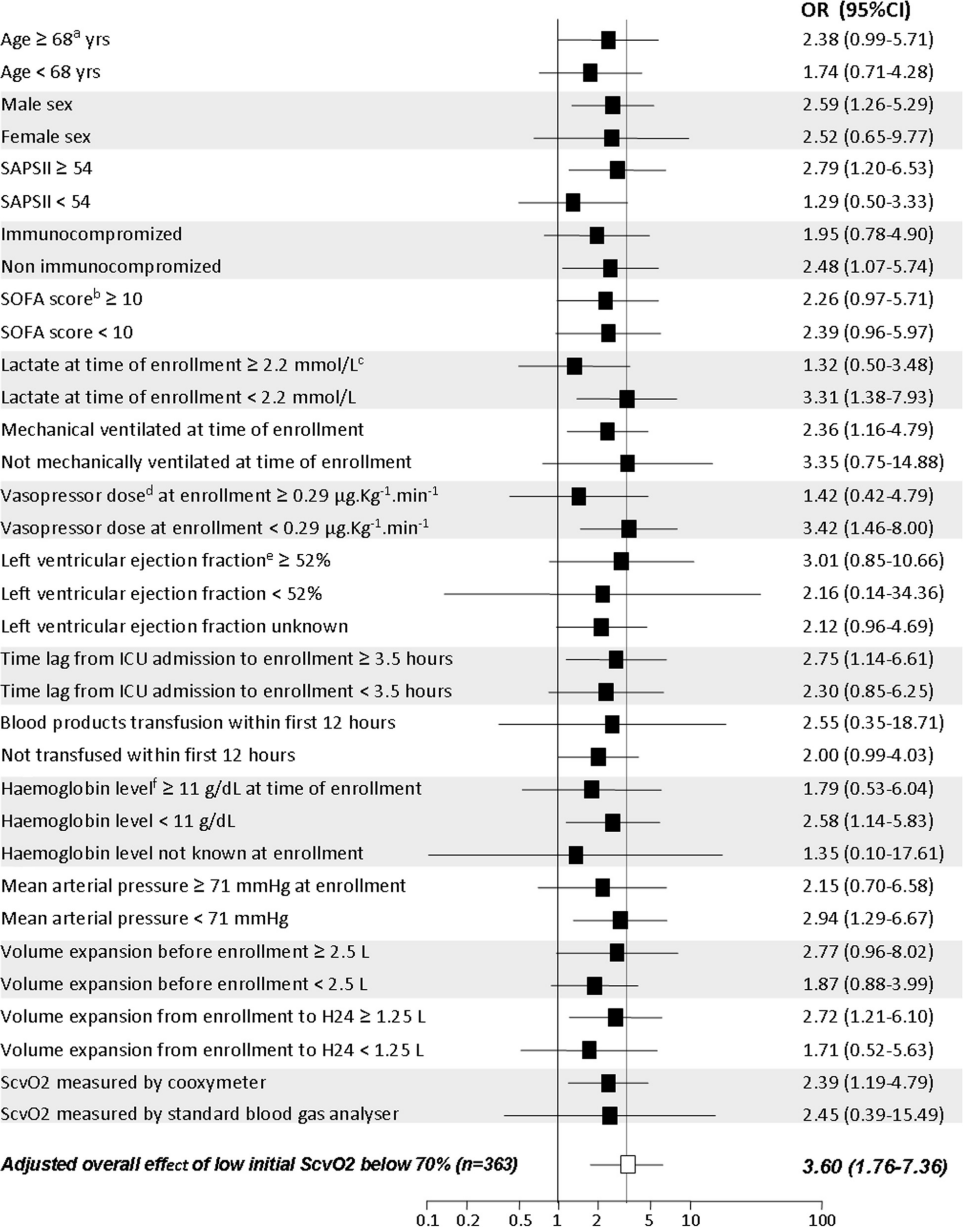
**Forest plot for subgroup analysis.** ^a^All cutoff values provided in the figure for demographic, clinical or laboratory variables are median values calculated on the whole study population. ^b^Sequential organ failure assessment (SOFA) score is the highest value during the first 24 hours after enrollment. ^c^With the use of a cutoff of 2 mmol/L for lactate, the odds ratio for day-28 death (OR) was 1.29 (0.54, 3.05) in the case of lactate >2 mmol/L, and 4.59 (1.79, 11.84) in the case of lactate ≤2 mmol/L. ^d^Vasopressor dose = continuous intravenous (iv) norepinephrine dose plus continuous iv epinephrine dose. ^e^Left ventricular ejection fraction assessed by transthoracic echocardiography before 24 hours after enrollment. ^f^Haemoglobin concentration was taken into account only if measured between 6 hours before and 6 hours after enrollment. SAPSII, Simplified acute physiology score; ScvO2, central venous oxygen saturation.

The initial S_cv_O_2_ entered in the logistic model as a continuous variable was negatively linked to day-28 mortality: OR = 0.96 (95% CI 0.93, 0.99) for each 1% increase in initial S_cv_O_2_; *P* = 0.004 (see Additional file 5: Table E9). An initial S_cv_O_2_ < 70% was significantly and independently associated with day-28 mortality (OR = 3.60, 95%CI 1.76, 7.36; *P* = 0.0004) (Table 3), a trend consistently observed across the different subgroups examined (Figure 2).

**Table 3:** **Logistic regression analysis of 28-day mortality in 363 septic patients, with initial S**_**cv**_ **O**_**2**_ **value below 70** ***%*** **adjusted for the other confounders**

**Covariables** ^**a**^	**Adjusted odds ratio** ^**b**^	**95% CI**	***P*** **-value**
SAPSII (for each 1 point increase)	1.05	1.03, 1.07	<0.00001
Initial S_cv_O_2_ < 70%	3. 60	1.76, 7.36	0.0004
Arterial lactate (for each 1 mmol/L increase)	1.18	1.06, 1.32	0.002
Initial arterial partial pressure in CO2 (for each 1 mmHg increase)	1.04	1.01, 1.06	0.003
McCabe class 1 (versus class 0)	2.58	1.31, 5.10	0.006
Abdominal sepsis	2.56	1.25, 5.23	0.010
McCabe class 2 (versus class 0)	3.09	1.24, 7.72	0.016
Male gender	2.14	1.11, 4.13	0.022
Initial body temperature (for each 1C° increase)	0.78	0.62, 0.98	0.031
Exposure to ACE inhibitors or ARB in the past 48 hours	0.50	0.26, 0.98	0.044

^a^All covariables entered in the model were variables linked to day-28 mortality with *P* <0.05 on univariate analysis, and selected using the backward method. ^b^For each continuous covariable odds ratios are given per each unit of the given covariable. ACE, angiotensin-converting enzyme; ARB, angiotensin II receptor blockers; SAPSII, Simplified acute physiology score; S_cv_O_2_, central venous oxygen saturation.

An initial S_cv_O_2_ < 75% was also significantly associated with day-28 mortality (OR = 2.15, 95% CI 1.16 to 3.98; *P* = 0.015) (see Additional file 5: Table E10).

### Evolution of S_cv_O_2_ and relationship to lactate concentration

The evolution of S_cv_O_2_ between H0 and H6 was similar between survivors and non survivors on day 28 (see Additional file 6: Figure E3). However, by logistic regression analysis, a S_cv_O_2_ < 70% at H6 still appeared as a risk factor for day-28 death (OR = 2.18, 95% CI 1.12, 4.26; *P* = 0.022) (see Additional file 5: Table E11).

**Figure 3:**
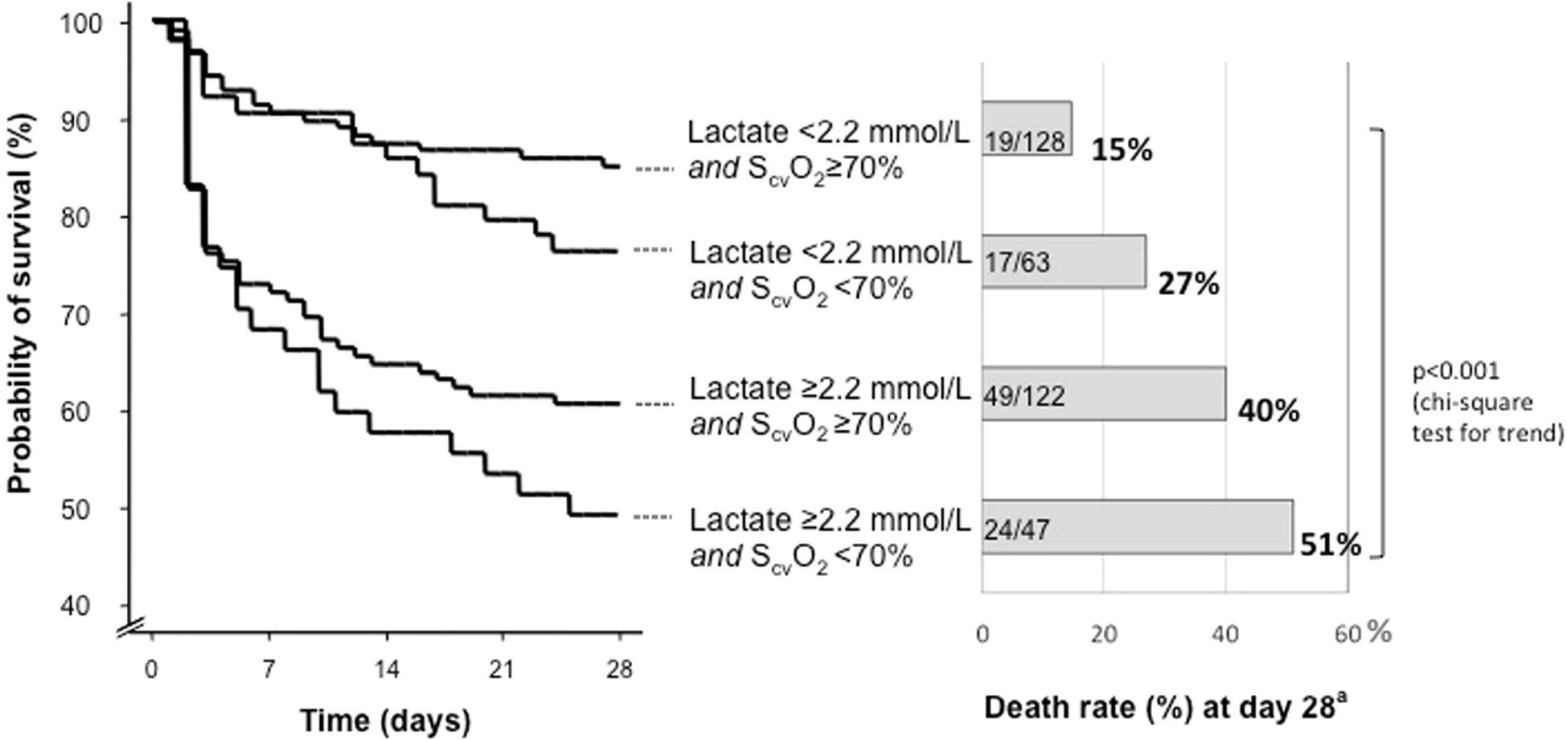
**Survival curve and death rate (%) at day 28 according to initial lactate level and central venous oxygen saturation (S**_**cv**_ **O**_**2**_ **).** The left part of the figure shows survival curves in four patients’ subgroups according to their initial zero hours (H0) lactate concentration and S_cv_O_2_. The right part of the figure shows the day-28 death rate in each subgroup. Numbers inside the bars are number of non survivors/total number of patients in each subgroup. ^a^Unadjusted pair comparison of survival curves between the different subgroups were not statistically significant on log-rank test. ^b^There was no significant difference in crude death rate at day-28 among the four groups (chi-squared test). However, there was a significant global trend towards higher death rate from the condition with normal lactate and S_cv_O_2_ to the condition with high lactate and low S_cv_O_2_ (*P* <0.001, Cochran-Armitage test).

The proportion of patients with initial S_cv_O_2_ < 70% was similar in the case of lactate above or below the median initial lactate value of 2.2 mmol/L (29% versus 33%; *P* = 0.3). Figure 3 shows the survival curves and the crude death rates at day 28 in four patients’ subgroups according to their lactate concentration and S_cv_O_2_ at H0 (for subgroups according to lactate and S_cv_O_2_ at H6 see Additional file 6: Figure E4). In subgroup analysis, the logistic regression showed that S_cv_O_2_ < 70% was independently associated with day-28 death in patients with initial lactate level <2.2 mmol/L (OR = 3.31) (Figure 2).

Among the 335 patients with lactate and S_cv_O_2_ measured at H0 and H6, 148 (44%) had a satisfactory lactate clearance (defined as at least a 10% decrease between H0 and H6 [25]) and 187 (56%) had not. The proportion of patients with S_cv_O_2_ < 70% at H6 was similar in the case of satisfactory or not satisfactory lactate clearance (22% versus 23%, respectively). Additional file 6: Figure E5 shows the survival curves and the crude death rates at day-28 in four patients’ subgroups according to their lactate clearance and S_cv_O_2_ at H0 and H6. On logistic regression, S_cv_O_2_ < 70% at H6 was independently associated with day-28 mortality in the subgroup of patients with non satisfactory lactate clearance (OR = 2.32 (1.03, 5.23)) but not in patients with satisfactory lactate clearance (OR = 0.98 (0.21, 4.54)). Additional results for the roles played by initial respiratory conditions, haemoglobin concentration and body temperature in the prevalence of low S_cv_O_2_ and mortality are given in Additional file 7.

## Discussion

The main finding of our study is that more than one quarter of septic patients had ScvO2 < 70% in the first hours after ICU admission. Furthermore, low ScvO2 in the first hours of ICU admission (and/or 6 hours later) was independently associated with 28-day mortality. These data suggest that one quarter of septic patients admitted to the ICU could be candidates for ICU protocol-based therapy targeting ScvO2.

### Prevalence of low ScvO2

In the present multicentre study, using ScvO2 measurements planned as early as possible in routine care, we report a 27% prevalence of low ScvO2 (<70%). This value is below the 40% to 50% prevalence reported in the few, small sized, available prospective studies performed to date in the ICU [9,10]. These studies analysed selected patients: one study analysed only half of the patients with lactate above 3 mmol/L enrolled in a comparative therapeutic trial [9], and the other study excluded patients with malignancy [10]. However, the 27% prevalence of low ScvO2 we observed in our study with few exclusion criteria represents a non negligible proportion of septic patients and does not support the view that low ScvO2 is uncommon after admission to the ICU [5-8].

Importantly, even when classic clinical endpoints of initial resuscitation were achieved (mean blood pressure (BP) >65 mmHg, central venous pressure (CVP) > 8 mmHg and urine output >0.5/ml/Kg) low ScvO2 could not be ruled out as it was observed in one third of our apparently resuscitated patients. Furthermore, arterial lactate also misclassified patients in their low/high contemporaneous ScvO2: the prevalence of ScvO2 < 70% was similar in the case of lactate above or below the median value of 2.2 mmol/L (29% versus 33%; *P* = 0.3). This underlines that ScvO2 measurement is not interchangeable with these routine clinical and biological data.

### Association between low ScvO2 and outcome

As expected, the SAPSII score and the arterial lactate consistently appeared as powerful predictors of day-28 death. Independently of these two well-known predictors [24,26], low initial ScvO2 was also consistently linked to day-28 mortality. To the best of our knowledge, this has never been previously shown in either emergency department or ICU studies. Of note, our study was not designed to assess whether the link between low ScvO2 and mortality is a causal relationship.

### Clinical perspectives

One quarter of our septic patients had low ScvO2 in the first hours of ICU admission and this was associated with poor outcome. This was also the case for persisting low ScvO2 (at 6 hours after the first measurement). These patients could be candidates for ICU protocol-based therapy targeting the ScvO2 in addition to routine initial care. The choice between lactate-guided, ScvO2-guided therapy, or between both, is still under debate [27]. In emergency department septic patients, there is no significant difference in patients’ outcomes when managed either with lactate-guided or ScvO2-guided therapy [28]. Interestingly, our results suggest that, rather than choosing one of these measurements, ScvO2 should be tested in combination with lactate monitoring because low ScvO2 may be present or persist even in the case of low lactate, or in the case of satisfactory lactate clearance (see Figure 3 and Additional file 6). This later finding was also observed in studies performed in emergency department patients in the very early phase of resuscitation: Arnold *et al*. [29] have shown that 15% of patients with good lactate clearance and 21% of patients with poor lactate clearance had S_cv_O_2_ < 70%; in the study by Jones *et al*. [28], an interventional, randomized trial comparing lactate clearance or S_cv_O_2_ > 70% as therapeutic targets, the proportion of patients who did not have normalized ScvO2 was 19% in both groups. This proportion is lower than the one we observed but may not reflect what happens in real conditions, as resuscitation was performed per protocol for the purpose of the randomized trial. For their part, Jansen *et al*. [9] compared usual care and protocol-based therapy with lactate clearance as a target in an ICU population of septic patients, and showed that at 6 hours, 33% of patients in the intervention group still had S_cv_O_2_ < 70% while more than 40% had lactate clearance >20%. Although these findings are consistent with ours, once again they may not reflect the real-life conditions as the patients were treated according to a rigorous and complex protocol. Therefore, to our knowledge, our study is the first to show that S_cv_O_2_ and lactate may show non parallel evolutions in septic patients cared for in the ICU in real-life conditions.

The usefulness of protocol-based resuscitation including the normalization of ScvO2 has been recently challenged by the ProCESS trial [30], a randomized trial conducted in emergency departments in the very early phase of resuscitation of septic patients. This study showed no advantage in terms of survival with the protocol-based therapy including the normalization of S_cv_O_2_ as compared to usual care [30]. Of note, the ProCESS trial population was quite different from that we studied in our work, in which we estimated the prevalence of S_cv_O_2_ and of persisting S_cv_O_2_ in ICU patients, after the initial phase of resuscitation. On the other hand, in 1995 Gattinoni *et al*. [31] also showed that therapy aimed at normalizing the SvO2 value did not lead to better outcome than standard care in a general population of critically ill patients. Their results cannot be transposed to our study population as they included fewer than 25% of septic patients, and included them more than 48 hours after ICU admission [31], a time frame quite different from that used for inclusion in our work. For these reasons we believe it is premature to abandon the concept of normalization of S_cv_O_2_ in severely septic patients admitted to the ICU for whom low S_cv_O_2_ cannot be neglected, given its association with increased day-28 mortality. Further studies are required to precisely identify which ICU patients would benefit from therapeutic strategies aimed at S_cv_O_2_ normalization.

The LOWESS plot (see Additional file 4) used to identify cutoff values of initial S_cv_O_2_ illustrates that below ScvO2 of 75%, the lower the S_cv_O_2_ the lower was the survival. Hence, S_cv_O_2_ < 75% was also associated with day-28 mortality in our multivariate analysis. This cutoff of 75% for worse outcome in our septic patients is higher than that found in cardiac failure patients [32,33]. This is consistent with the physiopathology of septic shock in which microcirculatory shunts lead to intra-organ oxygen extraction impairment that necessitates maintaining a high level of oxygen delivery to ensure adequate organ oxygen uptake [34]. Beyond the confirmation that S_cv_O_2_ of 70% is an important threshold for patient outcome, our study also suggests that, in septic patients, S_cv_O_2_ of 75% could be an even more relevant target that best ensures that overall tissue oxygenation is adequate in the context of septic oxygen extraction impairment. However, the thresholds of S_cv_O_2_ that could be proposed after the visual inspection of the LOWESS plot were statistically estimated cutoff values in the entire study population that may not apply at the individual level. Therefore, S_cv_O_2_ value of 67% for instance does not necessarily place a given patient in a dangerous condition, and conversely an S_cv_O_2_ of 73% should not be seen as necessarily reassuring. In fact each individual patient probably has his/her own threshold from which it could be dangerous to deviate, depending on how his/her tissues are trained to extract oxygen in situations of high oxygen demand [35] or insufficient oxygen transport, and depending on the intensity of the microcirculatory shunts [36] linked to the severity of the sepsis process. In relation with this later issue, high values of ScvO2 may reflect the intensity of the microcirculatory derangements caused by sepsis. Some studies have suggested that either ScvO2 higher than 80% within the first 3 days in the ICU [37] or ScvO2 higher than 90% in the emergency department [38], were markers of poor prognosis. Indeed, although our study was underpowered to specifically examine the role of high values of S_cv_O_2_, we also observed that, beyond the threshold of 75%, the higher the S_cv_O_2_, the lower was the survival (see Additional file 4).

### Limitations of the study

First, this study was not designed to seek for the reasons of low S_cv_O_2_. Since we have not systematically measured cardiac output, we cannot distinguish between the roles played by too-low oxygen delivery on the one hand and too-high oxygen demand on the other hand. Second, our study was clearly underpowered to draw conclusions about the significance of high S_cv_O_2_ values. Third, in view of our inclusion rate, selection bias cannot be definitely ruled out. However, patients were not selected on the basis of the variable of interest (S_cv_O_2_), which was not known during the screening/enrolment process. Additionally, enrolled and non enrolled patients’ characteristics were similar (see Additional file 2). Moreover, as shown in Additional file 2, the proportion of patients with low S_cv_O_2_ consistently ranged from around 24% to 36% in subgroups of patients classified according to their cardiac or respiratory status, or to the timing and intensity of therapeutic interventions performed before inclusion. Fourth, S_cv_O_2_ was not measured by the same method in all participating centres, as four used standard blood gas analysers and six used co-oximeters. This might have distorted the accurate determination of cutoff values of ScvO2, because the limits of agreement between both methods are wide [39]. However, this does not impair the general interpretation of our results as a majority (265/363) of the included patients had S_cv_O_2_ measured by co-oximetry and the link between low S_cv_O_2_ and day-28 mortality remained statistically significant in this patient subgroup.

## Conclusion

Our study demonstrated that, in septic patients, low levels of S_cv_O_2_ in the first hours of ICU admission were frequent (<70% for more than one quarter), even when clinical resuscitation endpoints are achieved and even when arterial lactate was normal. Importantly, S_cv_O_2_ in the first hours of ICU admission and/or six hours later was linked to day-28 mortality.

## Key messages

- In septic shock patients, low S_cv_O_2_ (<70%) is common in the first hours of admission to the ICU
- In septic shock patients, low S_cv_O_2_ (<70%) in the first hours of admission to the ICU is associated with increased day-28 mortality
- In addition to usual care, ICU protocol-based therapy targeting S_cv_O_2_ may be tested in controlled trials in septic patients with low or persisting low S_cv_O_2_ in the first hours of ICU admission

## Additional files

Additional file 1:
**Clinical and laboratory data recorded.**
Additional file 2:
**Comparison of enrolled and non enrolled patients and proportions of patients with low central venous oxygen saturation (S**_**cv**_ **O**_**2**_ **) in patients’ subgroups.**
Additional file 3:
**Inclusion delays and resuscitation before inclusion: influence on central venous oxygen saturation (S**_**cv**_ **O**_**2**_ **).**
Additional file 4:
**Association between central venous oxygen saturation (S**_**cv**_ **O**_**2**_ **) and day-28 survival as assessed by locally weighted scatterplot smoothing (LOWESS) plot.**
Additional file 5:
**Thresholds of central venous oxygen saturation (S**_**cv**_ **O**_**2**_ **) and mortality: bivariate and multivariate analyses.**
Additional file 6:
**Evolution of central venous oxygen saturation from hour-zero (H0)n to H6.**
Additional file 7:
**Roles of respiratory condition, haemoglobin, and body temperature.**

## Abbreviations

LOWESS: locally weighted scatterplot smoothing
OR: adjusted odds ratios
SAPSII: Simplified acute physiology score
S_cv_O_2_: central venous oxygen saturation
S_v_O_2_: mixed venous oxygen saturation
