# Supplementary material for: Prevalence of low central venous oxygen saturation in the first hours of intensive care unit admission and associated mortality in septic shock patients: a prospective multicentre study
Source: Crit Care. 2014 Nov 6;18(6):609. doi: 10.1186/s13054-014-0609-7 (PMC4265332; doi:10.1186/s13054-014-0609-7)
Supplement: Additional file 1: — Clinical and laboratory data recorded. [file 13054_2014_609_MOESM1_ESM.pdf]

Additional file 1

Title: Prevalence of low central venous oxygen saturation in the first hours of intensive care unit admission and associated mortality in septic shock patients: A prospective multicenter study.

Authors: Thierry Boulain, MD, Denis Garot, MD, Philippe Vignon, MD, Jean-Baptiste Lascarrou, MD, Arnaud Desachy, MD, Vlad Botoc, MD, Arnaud Follin, MD, Jean-Pierre Frat, MD, Frédéric Bellec, MD, Jean-Pierre Quenot, MD, PhD, Armelle Mathonnet, MD, Pierre-François Dequin, MD, PhD, Clinical Research in Intensive Care and Sepsis (CRICS) Group

Corresponding author : thierry.boulain@chr-orleans.fr

Data collection

The measurement of $S_{cv}O_2$ was performed as soon as possible (at the time defined as H0) by sampling blood from the superior vena cava through the central venous catheter, and then at the sixth hour (H6). $S_{cv}O_2$ was either calculated from blood gas analysis by a standard blood gas analyzer in four centers, or measured by a co-oximeter in six centers. Concomitantly, arterial blood was drawn at each time point for blood gas analysis and lactate measurement.

In addition to the planned central venous and arterial blood measurements, the following demographic, clinical and laboratory variables were prospectively recorded: age, gender, date and mode of admission (transfer from another ward or from the Emergency Department or direct admission), time of shock onset, severity of sepsis (severe sepsis or septic shock [1]), time of severe sepsis onset, Simplified Acute Physiology Score (SAPS) II [2] on admission, maximal Sepsis-related Organ Failure Assessment score (SOFA) [3] between H0 and H24, McCabe score [4], ICU mortality, mortality at day 28, origin of sepsis, type of sepsis acquisition (nosocomial or community-acquired), results of echocardiography if performed between admission and H24, exposure to angiotensin-converting enzyme inhibitors or angiotensin II receptor blockers within the two days preceding the onset of shock, underlying chronic diseases (chronic obstructive pulmonary disease, chronic respiratory failure, diabetes, chronic heart failure, past history of myocardial infarction, chronic hypertension, liver cirrhosis, chronic hemodialysis), and existence of immunosuppression.

Immunosuppression was defined as daily steroid therapy (prednisone-equivalent dose> 5mg/day) for more than 3 months, use of anti-rejection drugs, neutropenia (<1000 neutrophil/mm^3), active solid cancer, hematologic malignancy, radiotherapy or antitumoral chemotherapy in the past 6 months, or HIV seropositivity.

References

1. Levy MM, Fink MP, Marshall JC, Abraham E, Angus D, Cook D, Cohen J, Opal SM, Vincent JL, Ramsay G: **2001 SCCM/ESICM/ACCP/ATS/SIS International Sepsis Definitions Conference**. *Crit Care Med* 2003, **31**:1250-1256.

2. Le Gall JR, Lemeshow S, Saulnier F: **A new Simplified Acute Physiology Score (SAPS II) based on a European/North American multicenter study**. *JAMA* 1993, **270**: 2957-63.

3. Vincent JL, de Mendonça A, Cantraine F, Moreno R, Takala J, Suter PM, Sprung CL, Colardyn F, Blecher S: **Use of the SOFA score to assess the incidence of organ dysfunction/failure in intensive care units: results of a multicenter, prospective study. Working group on "sepsis-related problems" of the European Society of Intensive Care Med**. *Crit Care Med* 1998, **26**:1793–1800.

4. McCabe WR, Jackson GG: **Gram-negative bacteremia. I. Etiology and ecology.** *Arch Intern Med* 1962, **110**:847–855.
