# Supplementary material for: Prevalence of low central venous oxygen saturation in the first hours of intensive care unit admission and associated mortality in septic shock patients: a prospective multicentre study
Source: Crit Care. 2014 Nov 6;18(6):609. doi: 10.1186/s13054-014-0609-7 (PMC4265332; doi:10.1186/s13054-014-0609-7)
Supplement: Additional file 2: — Comparison of enrolled and non enrolled patients and proportions of patients with low central venous oxygen saturation (S cv O 2 ) in patients’ subgroups. [file 13054_2014_609_MOESM2_ESM.pdf]

Additional file 2

Title: Prevalence of low central venous oxygen saturation in the first hours of intensive care unit admission and associated mortality in septic shock patients: A prospective multicenter study

Authors: Thierry Boulain, MD, Denis Garot, MD, Philippe Vignon, MD, Jean-Baptiste Lascarrou, MD, Arnaud Desachy, MD, Vlad Botoc, MD, Arnaud Follin, MD, Jean-Pierre Frat, MD, Frédéric Bellec, MD, Jean-Pierre Quenot, MD, PhD, Armelle Mathonnet, MD, Pierre-François Dequin, MD, PhD, Clinical Research in Intensive Care and Sepsis (CRICS) Group

Corresponding author : thierry.boulain@chr-orleans.fr

Comparison of enrolled and non enrolled patients

Among the 670 screened patients, the SAPSII was not different between eligible and non eligible patients (56.6 ± 20.4 vs 53.6 ± 21.9, respectively; p=0.07), or between enrolled and not enrolled patients (56.8 ± 19.9 vs 54.1 ± 22.0, respectively; p=0.10).

Among the 670 screened patients, 76 out of 234 (32%) non eligible patients versus 128/436 (29%) eligible patients died in the ICU (p=0.43), and 102/307 (33%) non enrolled patients versus 102/ 363 (28%) enrolled patients died in the ICU (p=0.43). When adjusted for center as a random effect in a mixed effect logistic regression model, the SAPSII was strongly linked to ICU death, whereas status regarding the potential eligibility in the study or regarding the inclusion or not, were not significantly associated with ICU death (Table E1).

Table E1 : Results of logistic regression with death in the Intensive Care Unit as the dependant variable among the 670 screened patients

	p-value	OR	95%CI
SAPSII *for each 1 point-increase*	$<1.10^{-10}$	1.046	1.036 – 1.056
Eligible patient/not eligible patient	0.93	1.028	0.556 – 1.898
Enrolled/not enrolled patient	0.16	0.654	0.362 – 1.185

Among the 436 eligible patients, 26/73 (36%) non enrolled patients versus 102/363 (28%) enrolled patients died in the ICU (p=0.21). In a mixed effect logistic regression model, the status regarding the inclusion or not was not significantly associated with ICU mortality when adjusted for center as a random effect (Table E2).

Table E2 : Results of logistic regression with death in the Intensive Care Unit as the dependant variable among the 436 eligible patients

	p-value	OR	95%CI
SAPSII *for each 1 point-increase*	$<1.10^{-10}$	1.046	1.033 – 1.059
Enrolled/not enrolled patient	0.14	0.638	0.351 – 1.159

Relationship between the prevalence of low ScvO2 (<70%) and the ratio enrolled upon screened patients in each centre.

Despite a high number of patients were missed for inclusion in our study cohort, Figure E1 shows that there was no obvious relationship between the rate of missed patients and the prevalence of low (<70%) ScvO2 in each centre.

Figure E1:

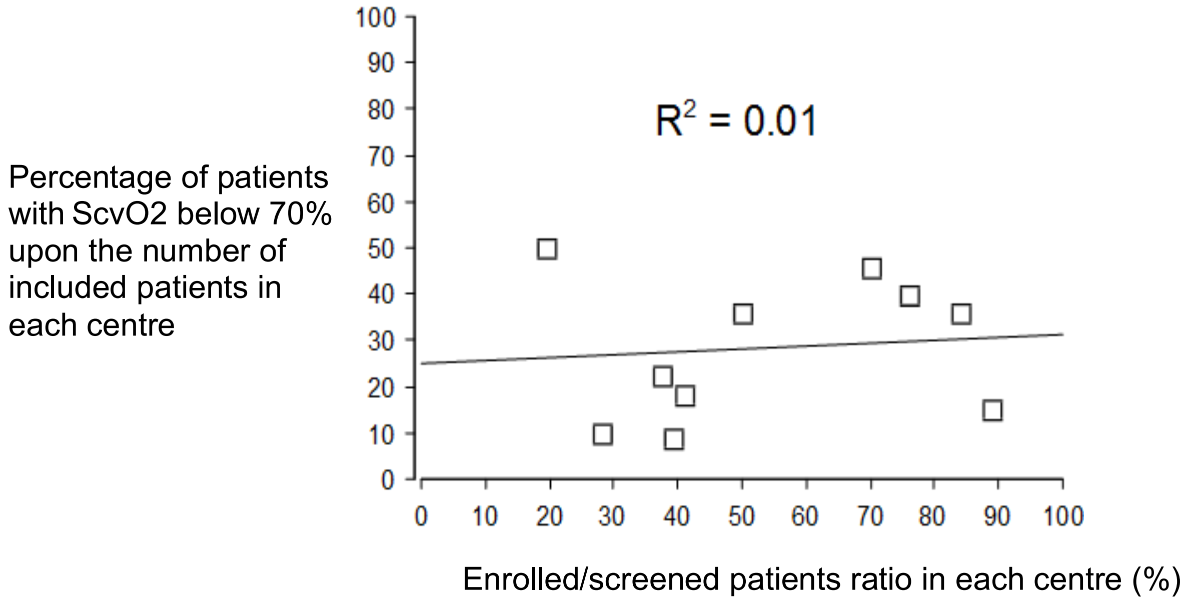

Prevalence of low (< 70%) ScvO2 in subgroups defined *a posteriori*

As illustrated in the following Tables (Tables E3 to E6), the proportion of patients with low ScvO2 was consistently (with very few exceptions) around 24-36% whatever the subgroups considered. This suggests that if discrepancies may have exist between the source population and the actual study population concerning the patients' repartition into the different subgroups, these discrepancies were not large enough to lead to a significantly biased estimate of the prevalence of ScvO2 below 70%.

Table E3: Proportion of patients with ScvO2 below 70% in different categories of patients classified according to the sum of their cardiovascular risk factors[a].

Sum of factors	Number of patients in each category	Number of patients with ScvO2 below 70%	Proportion (%) of patients with ScvO2 below 70%	95% confidence interval of the proportion
0	123	37	**30.1**	22 - 39
1	155	46	**29.7**	23 - 38
≥ 2	85	28	**32.9**	23 - 44

[a]: For the purpose of this classification, risk factors taken into account were : existence of chronic cardiac failure, chronic arterial hypertension, permanent atrial fibrillation, and known left ventricular ejection fraction below 50%.

Table E4: Proportion of patients with ScvO2 below 70% in patients with different respiratory status

	Number of patients in each category	Patients with ScvO2 <70%	Proportion (%) of patients with ScvO2 <70%	95% confidence interval of the proportion
No COPD	300	90	**30.0**	25 - 36
COPD	63	21	**33.3**	22 - 46
No Chronic respiratory disease	316	96	**30.4**	25 - 36
Chronic respiratory disease	47	15	**31.9**	20 - 47

COPD: Chronic obstructive pulmonary disease

Table E5: Proportion of patients with ScvO2 below 70% according to the time elapsed between sepsis identification, or admission, or presence of inclusion criteria, and inclusion.

	N	Patients with ScvO2 <70%	Proportion (%) of patients with ScvO2 <70%	95% confidence interval of the proportion
**Time elapsed between severe sepsis identification and inclusion**				
1rst quintile (median:150 min)	72	18	**25.0**	15 – 37
2nd quintile (median:282 min)	74	18	**24.3**	15 – 36
3rd quintile (median:455 min)	71	23	**32.4**	22 – 45
4th quintile (median:746 min)	73	26	**35.6**	25 – 48
5th quintile (median:1440 min)	73	26	**35.6**	25 - 48
**Time elapsed between ICU admission and inclusion**				
1rst quintile (median:86 min)	72	26	**36.1**	25 – 48
2nd quintile (median:150min)	73	23	**31.5**	21 – 44
3rd quintile (median:211 min)	74	21	**28.4**	19 – 40
4th quintile (median:345 min)	71	23	**32.4**	22 – 45
5th quintile (median:755 min)	73	18	**24.7**	16 – 36
**Time elapsed between onset of inclusion criteria and inclusion**				
1rst quintile (median:86 min)	73	16	**21.9**	13 – 33
2nd quintile (median:150min)	82	28	**34.1**	24 – 46
3rd quintile (median:211 min)	73	22	**30.1**	20 – 42
4th quintile (median:345 min)	74	25	**33.8**	23 – 46
5th quintile (median:755 min)	61	20	**32.8**	22 – 46

Table E6: Proportion of patients with ScvO2 below 70% according to the amount of fluids received for resuscitation before inclusion, and according to the norepinephrine dosage administered at inclusion

	N	Patients with ScvO2 <70%	Proportion (%) of patients with ScvO2 <70%	95% confidence interval of the proportion
**Amounts of fluids for resuscitation before inclusion (mL/Kg of body weight)**				
1rst quintile (median: 11 mL/Kg)	72	17	**23.6**	15 – 35
2nd quintile (median: 25 mL/Kg)	72	23	**31.9**	22 – 44
3rd quintile (median: 36 mL/Kg)	72	29	**40.3**	29 – 53
4th quintile (median: 53 mL/Kg)	72	17	**23.6**	15 – 35
5th quintile (median: 79 mL/Kg)	75	25	**33.3**	23 - 45
**Dosage of continuous iv norepinephrine administered at time of inclusion (µg/Kg/min)**				
1rst quintile (median: 0 µg/Kg/min)	53	21	**39.6**	27 – 54
2nd quintile (median: 0.125 µg/Kg/min)	76	28	**36.8**	26 – 49
3rd quintile (median: 0.250 µg/Kg/min)	78	23	**29.5**	20 – 41
4th quintile (median: 0.435 µg/Kg/min)	80	17	**21.3**	13 – 32
5th quintile (median: 0.905 µg/Kg/min)	76	22	**28.9**	19 – 41

Table E7: Proportion of ScvO2 below 70% according to the patients' origin.

	N	Patients with ScvO2 <70%	Proportion (%) of patients with ScvO2 <70%	95% confidence interval of the proportion
Transferred from another hospital	93	28	**30.1**	21 – 41
Transferred from the ward	95	30	**31.6**	23 – 42
Transferred from the emergency department	175	53	**30.3**	24 – 38
