# Supplementary material for: Prevalence of low central venous oxygen saturation in the first hours of intensive care unit admission and associated mortality in septic shock patients: a prospective multicentre study
Source: Crit Care. 2014 Nov 6;18(6):609. doi: 10.1186/s13054-014-0609-7 (PMC4265332; doi:10.1186/s13054-014-0609-7)
Supplement: Additional file 3: — Inclusion delays and resuscitation before inclusion: influence on central venous oxygen saturation (S cv O 2 ). [file 13054_2014_609_MOESM3_ESM.pdf]

Additional file 3

Title: Prevalence of low central venous oxygen saturation in the first hours of intensive care unit admission and associated mortality in septic shock patients: A prospective multicenter study.

Authors: Thierry Boulain, MD, Denis Garot, MD, Philippe Vignon, MD, Jean-Baptiste Lascarrou, MD, Arnaud Desachy, MD, Vlad Botoc, MD, Arnaud Follin, MD, Jean-Pierre Frat, MD, Frédéric Bellec, MD, Jean-Pierre Quenot, MD, PhD, Armelle Mathonnet, MD, Pierre-François Dequin, MD, PhD, Clinical Research in Intensive Care and Sepsis (CRICS) Group

Corresponding author : thierry.boulain@chr-orleans.fr

Inclusion delays and resuscitation before inclusion : influence on $S_{cv}O_2$

Mean initial $S_{cv}O_2$ values were not different (all p-values > 0.2 by analysis of variance) between deciles of delay from ICU admission and inclusion, between deciles of delay from inclusion criteria occurrence and inclusion, and between deciles of delay from severe sepsis onset and inclusion. The proportions of patients with initial $S_{cv}O_2$ below 70% were also not different among the above decile groups (all p-values > 0.05).

The time elapsed between ICU admission and inclusion, or between the presence of criteria for inclusion and inclusion, or between severe sepsis onset and inclusion, was not different between patients with initial $S_{cv}O_2$ above or below 70% (all p>0.12). However, one half of patients were included 7.6 hours (median time) or more after the onset of severe sepsis (including 17% after the 24th hour) and these patients admitted late in the history of severe sepsis had more frequently a low initial $S_{cv}O_2$ (<70%) than patients included earlier (35.7% *vs.* 25.4%, respectively; p=0.04).

Mean initial $S_{cv}O_2$ values were not different (p-value > 0.05 by analysis of variance) between deciles of amount of vascular volume expansion received before inclusion. The amount of vascular volume expansion received within 24 h prior inclusion was similar in patients with initial $S_{cv}O_2$ above and below 70% (2.8 ±1.7 L for both, p=0.91). Initial $S_{cv}O_2$ values were similar in patients who received less or more than 2.5 L (median value) of volume expansion before inclusion (73.4±11.7% *vs.* 74.8%±10.0%, respectively; p=0.20). Proportions of patients with initial $S_{cv}O_2$ value below 70% were not different between the two subsets (34% *vs.* 27%, respectively; p=0.17).

Dosage of vasopressors (sum of continuous iv epinephrine and norepinephrine dosages) the patients were receiving at H0 tended to be slightly lower when initial $S_{cv}O_2$ was below 70% (0.36±0.04 *vs.* 0.47±0.03 $\mu g.Kg^{-1}.min^{-1}$; p=0.066). The prevalence of low initial $S_{cv}O_2$ (<70%) was higher (p=0.003) in patients with high vasopressor doses (above 0.29$\mu g.Kg^{-1}.min^{-1}$ [median value]) at inclusion (38%) than in patients with lower vasopressor doses (23%). These patients with high doses of

vasopressors at inclusion had also a higher mortality at day 28 (38% *vs.* 24%; p=0.004).
