# Supplementary material for: Prevalence of low central venous oxygen saturation in the first hours of intensive care unit admission and associated mortality in septic shock patients: a prospective multicentre study
Source: Crit Care. 2014 Nov 6;18(6):609. doi: 10.1186/s13054-014-0609-7 (PMC4265332; doi:10.1186/s13054-014-0609-7)
Supplement: Additional file 4: — Association between central venous oxygen saturation (S cv O 2 ) and day-28 survival as assessed by locally weighted scatterplot smoothing (LOWESS) plot. [file 13054_2014_609_MOESM4_ESM.pdf]

Additional file 4

Title: Prevalence of low central venous oxygen saturation in the first hours of intensive care unit admission and associated mortality in septic shock patients: A prospective multicenter study.

Authors: Thierry Boulain, MD, Denis Garot, MD, Philippe Vignon, MD, Jean-Baptiste Lascarrou, MD, Arnaud Desachy, MD, Vlad Botoc, MD, Arnaud Follin, MD, Jean-Pierre Frat, MD, Frédéric Bellec, MD, Jean-Pierre Quenot, MD, PhD, Armelle Mathonnet, MD, Pierre-François Dequin, MD, PhD, Clinical Research in Intensive Care and Sepsis (CRICS) Group

Corresponding author : thierry.boulain@chr-orleans.fr

Association between $S_{cv}O_2$ and day-28 survival as assessed by LOWESS plot

The $S_{cv}O_2$ below 70% and other thresholds of initial $S_{cv}O_2$ values determined by locally weighted scatterplot smoothing (LOWESS) plot were examined for their potential association with day-28 mortality.

Figure E2: Locally weighted scatterplot smoothing (LOWESS) plot of the association of day-28 survival probability and initial central venous oxygen saturation value (%).

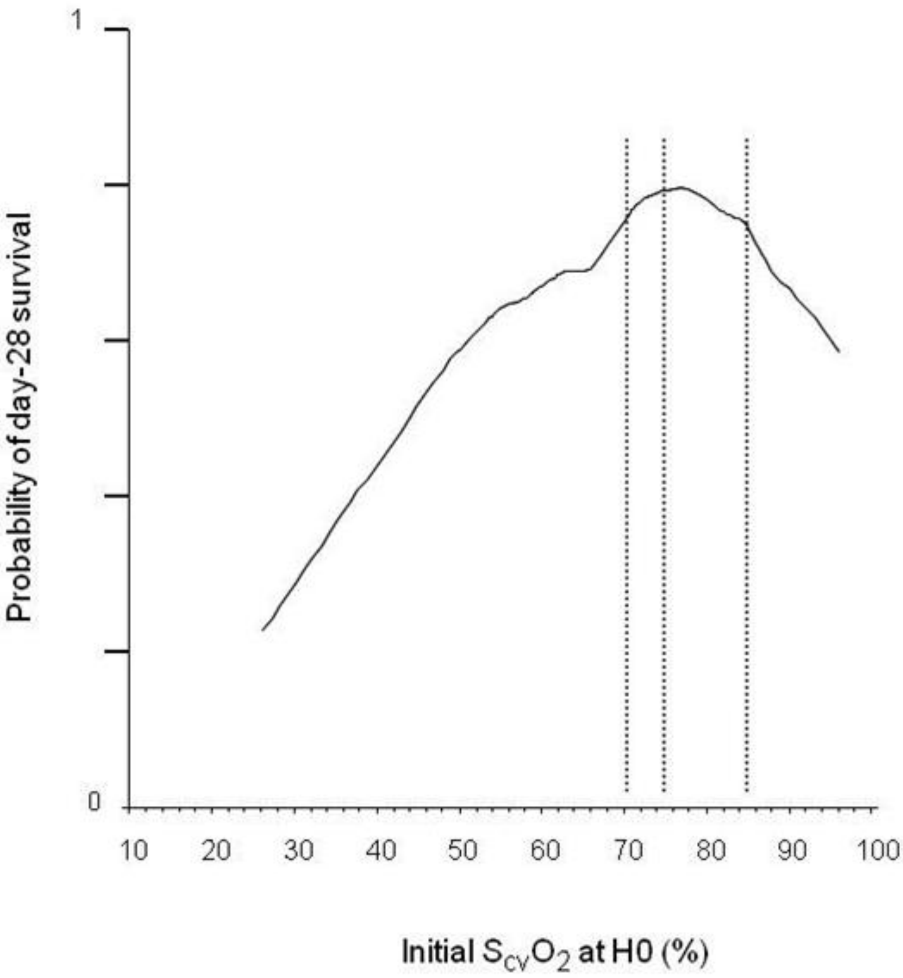

Legend: Vertical dashed lines indicate which thresholds of central venous oxygen saturation value were visually chosen to be examined for their potential association with day-28 mortality by univariate and multivariate analyses.
