# Supplementary material for: Prevalence of low central venous oxygen saturation in the first hours of intensive care unit admission and associated mortality in septic shock patients: a prospective multicentre study
Source: Crit Care. 2014 Nov 6;18(6):609. doi: 10.1186/s13054-014-0609-7 (PMC4265332; doi:10.1186/s13054-014-0609-7)
Supplement: Additional file 5: — Thresholds of central venous oxygen saturation (S cv O 2 ) and mortality: bivariate and multivariate analyses. [file 13054_2014_609_MOESM5_ESM.pdf]

Additional file 5

Title: Prevalence of low central venous oxygen saturation in the first hours of intensive care unit admission to the intensive care unit and associated mortality in septic shock patients: A prospective multicenter study.

Authors: Thierry Boulain, MD, Denis Garot, MD, Philippe Vignon, MD, Jean-Baptiste Lascarrou, MD, Arnaud Desachy, MD, Vlad Botoc, MD, Arnaud Follin, MD, Jean-Pierre Frat, MD, Frédéric Bellec, MD, Jean-Pierre Quenot, MD, PhD, Armelle Mathonnet, MD, Pierre-François Dequin, MD, PhD, Clinical Research in Intensive Care and Sepsis (CRICS) Group

Corresponding author : thierry.boulain@chr-orleans.fr

Thresholds of ScvO2 and mortality : bivariate and multivariate analyses

Table E8: Association of central venous oxygen saturation below or above different thresholds and death rate at day 28 in 363 patients with severe sepsis or septic shock

	Survivors at day 28, n (%)	Nonsurvivors at day 28, n (%)	p-value (Fisher exact test)
initial $S_{cv}O_2$ < 70%	69 (62.2)	42 (37.8)	
initial $S_{cv}O_2$ ≥ 70%	183 (72.6)	69 (27.4)	0.049
initial $S_{cv}O_2$ < 75%	118 (67.0)	58 (33.0)	
initial $S_{cv}O_2$ ≥ 75%	134 (71.7)	53 (28.3)	0.36
initial $S_{cv}O_2$ < 85%	225 (70.1)	96 (29.9)	
initial $S_{cv}O_2$ ≥ 85%	27 (64.3)	15 (35.7)	0.48
initial $S_{cv}O_2$ < 70% or ≥ 85%	96 (62.7)	57 (37.3)	
initial $S_{cv}O_2$ ≥ 70% and < 85%	156 (74.3)	54 (25.7)	0.02

Definition of abbreviations: initial $S_{cv}O_2$= central venous oxygen saturation at time of inclusion

Table E9: Logistic regression for 28-day mortality in 363 septic patients, with $S_{cv}O_2$ *value entered as a continuous variable*

Covariables	Adjusted Odds-ratio[a]	95% confidence interval	p-value
SAPS II (for each 1 point-increase)	1.05	1.03-1.07	<0.00001
Initial arterial lactate (for each 1mmol/L-increase)	1.17	1.05-1.30	0.004
Initial $S_{cv}O_2$ (for each 1%-increase)[b]	0.96	0.93-0.99	0.004
McCabe class 1 (*versus* class 0)	2.5	1.28-4.87	0.007
McCabe class 2 (*versus* class 0)	3.27	1.35-7.96	0.009
Initial arterial partial pressure in CO2 (for each 1mmHg-increase)	1.03	1.01-1.05	0.012
Male gender	2.09	1.08-4.02	0.028
Initial body temperature (for each 1°C-increase)	0.79	.64-.99	0.045
Abdominal sepsis	1.98	.98-4.00	0.057
Urinary tract infection	0.28	.07-1.16	0.079

Definition of abbreviations: SAPS II = Simplified acute physiology score; $S_{cv}O_2$ = Central venous oxygen saturation

[a] : For each continuous covariable Odds-ratios are given per each unit of the given covariable

[b]: The variable “initial $S_{cv}O_2$“ (continuous variable) was forced in the model. All the other covariables were variables linked to day-28 mortality with p<0.05 by univariate analysis and selected using the backward method.

Table E10: Logistic regression for 28-day mortality in 363 septic patients with *ScvO2 value at H0 below 75%* adjusted for the other confounders

Covariables	Adjusted Odds-ratio[a]	95% confidence interval	p-value
SAPS II (for each 1 point-increase)	1.05	1.03-1.07	<0.00001
Arterial lactate (for each 1mmol/L-increase)	1.16	1.06-1.27	0.002
McCabe class 2	3.34	1.48-7.55	0.004
Initial arterial partial pressure in CO2 (for each 1mmHg-increase)	1.03	1.01-1.05	0.005
Initial $S_{cv}O_2$ < 75% [b]	2.15	1.16-3.98	0.015
McCabe class 1	2.20	1.16-4.15	0.015
Liver cirrhosis	3.44	1.26-9.39	0.016
Urinary tract infection	0.26	0.08-0.93	0.038

Definition of abbreviations: SAPS II = Simplified acute physiology score; $S_{cv}O_2$ = Central venous oxygen saturation

[a] : For each continuous covariable odds-ratios are given per each unit of the given covariable

[b]: The variable "Initial $S_{cv}O_2$ < 75%" was forced in the model. All the other covariables were variables linked to day-28 mortality with p<0.05 by univariate analysis and selected using the backward method.

Table E11: Logistic regression for 28-day mortality in 345 septic patients, with $S_{cv}O_2$ *value at H6 below 70%* adjusted for SAPS II, McCabe classification and initial arterial lactate level

Covariables	Adjusted Odds-ratio[a]	95% confidence interval	p-value
$S_{cv}O_2$ at H6 <70%[b]	2.18	1.12-4.26	0.022
McCabe class 1 (*versus* class 0)	2.13	1.13-4.00	0.020
Arterial lactate (for each 1mmol/L-increase)	1.13	1.03-1.23	0.008
McCabe class 2 (*versus* class 0)	3.24	1.46-7.20	0.004
SAPS II (for each 1 point-increase)	1.05	1.03-1.06	<0.0001

Definition of abbreviations: SAPS II = Simplified acute physiology score; $S_{cv}O_2$ = Central venous oxygen saturation

[a]: For each continuous covariable odds-ratios are given per each unit of the given covariable.

[b]: The variable "$S_{cv}O_2$ at H6<70%" was forced in the model
