# Supplementary material for: Prevalence of low central venous oxygen saturation in the first hours of intensive care unit admission and associated mortality in septic shock patients: a prospective multicentre study
Source: Crit Care. 2014 Nov 6;18(6):609. doi: 10.1186/s13054-014-0609-7 (PMC4265332; doi:10.1186/s13054-014-0609-7)
Supplement: Additional file 6: — Evolution of central venous oxygen saturation from hour-zero (H0)n to H6. [file 13054_2014_609_MOESM6_ESM.pdf]

Additional file 6

Title: Prevalence of low central venous oxygen saturation in the first hours of intensive care unit admission and associated mortality in septic shock patients: A prospective multicenter study.

Authors: Thierry Boulain, MD, Denis Garot, MD, Philippe Vignon, MD, Jean-Baptiste Lascarrou, MD, Arnaud Desachy, MD, Vlad Botoc, MD, Arnaud Follin, MD, Jean-Pierre Frat, MD, Frédéric Bellec, MD, Jean-Pierre Quenot, MD, PhD, Armelle Mathonnet, MD, Pierre-François Dequin, MD, PhD, Clinical Research in Intensive Care and Sepsis (CRICS) Group

Corresponding author : thierry.boulain@chr-orleans.fr

Evolution of central venous oxygen saturation from H0 to H6

Figure E3:

Evolution of central venous oxygen saturation from H0 to H6

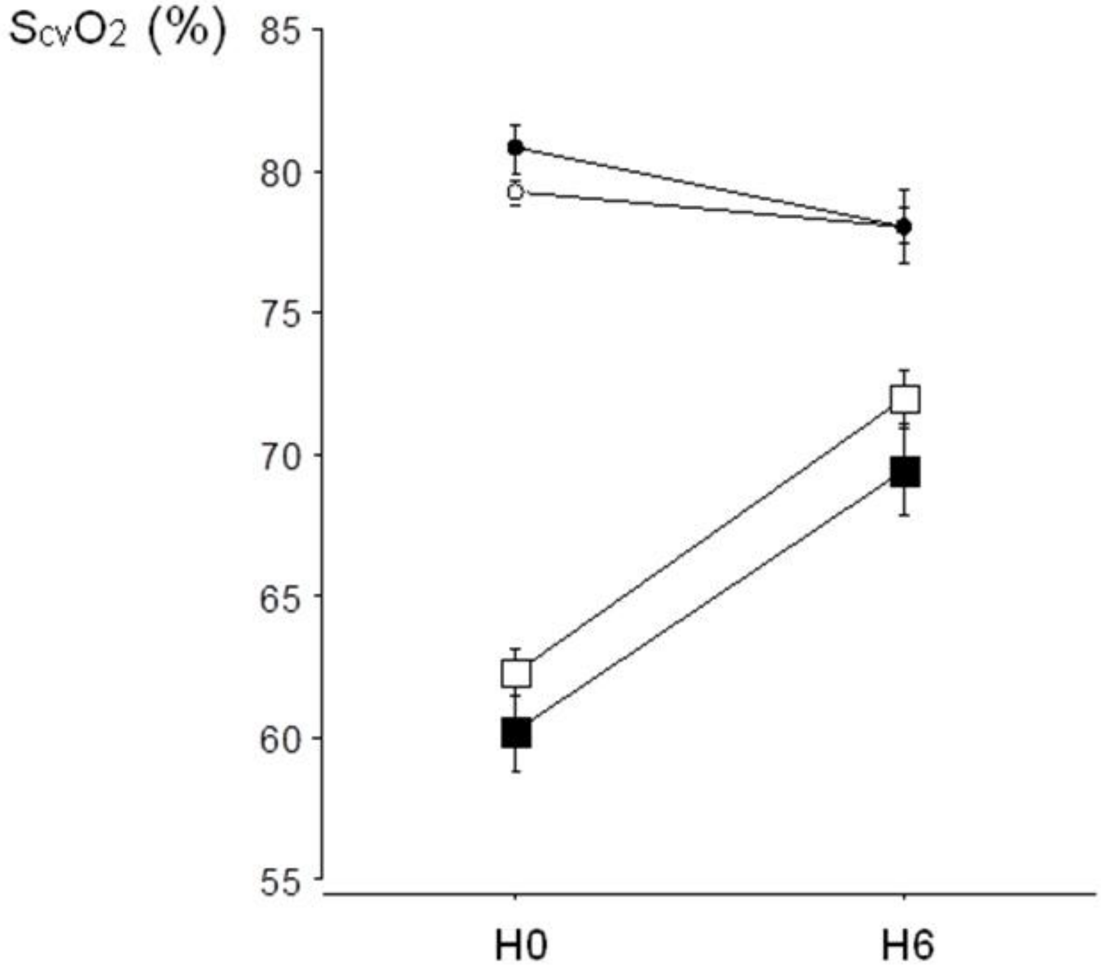

Legend: *Definition of abbreviations*: ScvO2 = central venous oxygen saturation; Circles represent patients with ScvO2 at H0 above 70%; Squares represent patients with ScvO2 at H0 below 70%; Empty and black symbols represent survivors and non-survivors at day 28, respectively. Bars represent standard errors.

Figure E4:

Survival curve and death rate (%) at day 28 according to lactate level and $S_{cv}O_2$ at the *sixth hour*

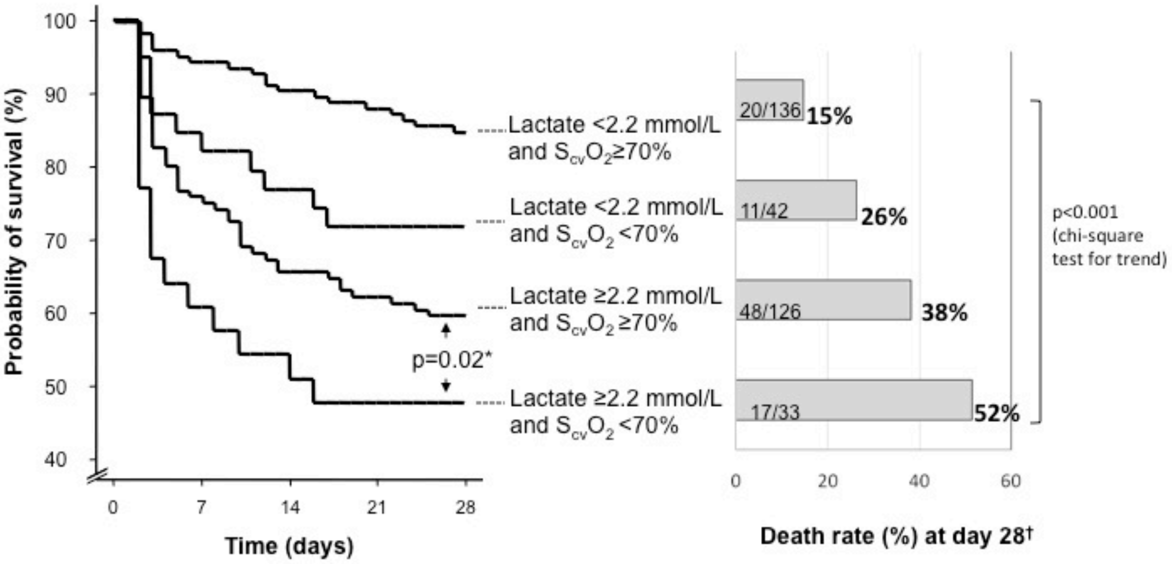

Legend: *Definition of abbreviations*: ScvO2 = central venous oxygen saturation; The left part of the figure shows survival curves in 4 patients' subgroups according to their lactate concentration and $S_{cv}O_2$ at H6. The right part of the figure shows the day-28 death rate in each subgroup. Numbers inside the bars are number of nonsurvivors/total number of patients in each subgroup.

[a]: Unadjusted pair comparison of survival curves reached statistical significance between $S_{cv}O_2$ below or above 70% at H6 in the subset of patients with initial lactate level above 2.2 mmol/L (p=0.02 by log rank test).

[b]: There was no significant difference in crude death rate at day-28 among the 4 groups (chi-squared test).. However, there was a significant global trend towards higher death rate from the condition with normal lactate and $S_{cv}O_2$ to the condition with high lactate and low $S_{cv}O_2$ (p<0.001 by Cochran-Armitage test).

Figure E5:

Survival curve and death rate (%) at day 28 according to lactate clearance and $S_{cv}O_2$ *at the sixth hour*

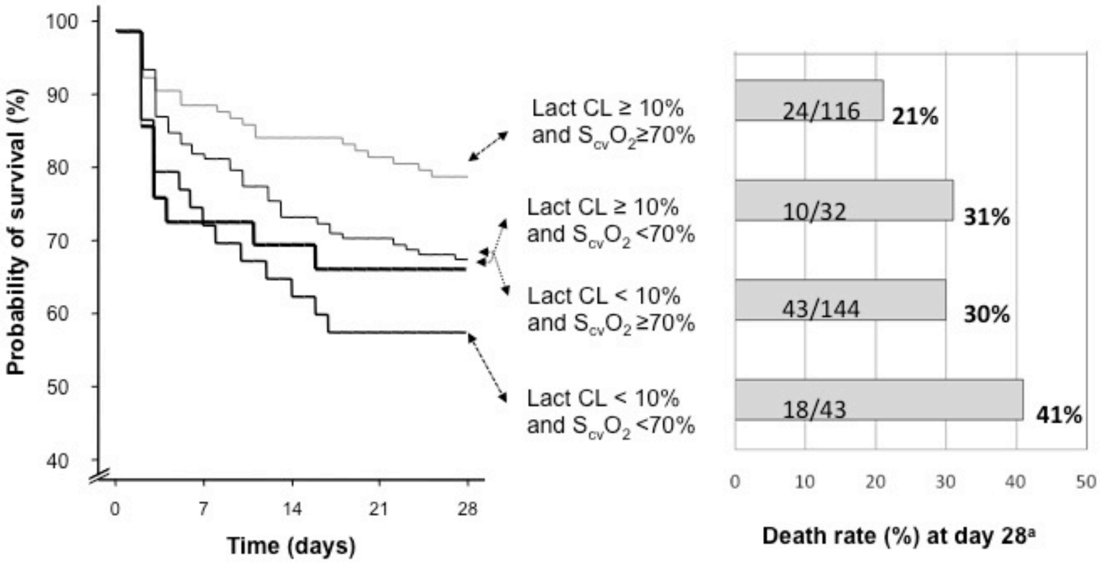

Legend:

When classifying the patients in 4 groups according to their lactate clearance (below or above 10%) and to their $S_{cv}O_2$ at H6 (below or above 70%), we observed no significant difference in pair comparisons of survival curves.

[a]: There was no significant difference in crude death rate at day-28 among the 4 groups (chi-squared test). However, there was a significant global trend towards higher death rate from the condition with good lactate clearance and $S_{cv}O_2 \geq 70\%$ at H6 to the condition with bad lactate clearance and low $S_{cv}O_2$ ($p=0.011$ by Cochran-Armitage test).
