# Supplementary material for: Prevalence of low central venous oxygen saturation in the first hours of intensive care unit admission and associated mortality in septic shock patients: a prospective multicentre study
Source: Crit Care. 2014 Nov 6;18(6):609. doi: 10.1186/s13054-014-0609-7 (PMC4265332; doi:10.1186/s13054-014-0609-7)
Supplement: Additional file 7: — Roles of respiratory condition, haemoglobin, and body temperature. [file 13054_2014_609_MOESM7_ESM.pdf]

Additional file 7

Title: Prevalence of low central venous oxygen saturation in the first hours of intensive care unit admission and associated mortality in septic shock patients: A prospective multicenter study.

Authors: Thierry Boulain, MD, Denis Garot, MD, Philippe Vignon, MD, Jean-Baptiste Lascarrou, MD, Arnaud Desachy, MD, Vlad Botoc, MD, Arnaud Follin, MD, Jean-Pierre Frat, MD, Frédéric Bellec, MD, Jean-Pierre Quenot, MD, PhD, Armelle Mathonnet, MD, Pierre-François Dequin, MD, PhD, Clinical Research in Intensive Care and Sepsis (CRICS) Group

Corresponding author : thierry.boulain@chr-orleans.fr

Roles of respiratory condition, haemoglobin, and body temperature

Role of initial respiratory condition

Initial arterial partial pressure of oxygen (P_aO_2) and $S_{cv}O_2$ were positively albeit weakly correlated (r^2=0.13; p<0.0001). The median initial PaO2 was 98 mmHg (IQR: 79-138). Patients with initial P_aO_2 below the median value had a higher prevalence of $S_{cv}O_2$ value below 70% than patients with PaO2 above or equal to median value (40% [72/179] *vs.* 21% [39/184], respectively; p<0.0001). The one hundred patients who were breathing spontaneously at H0 had a lower P_aO_2 (100±38 mmHg) than the 263 patients already intubated (129±77 mmHg) (p=0.0003). Spontaneously breathing patients had more frequently an initial $S_{cv}O_2$ value below 70% compared to already intubated patients (47% *vs.* 24%, respectively; p=0.0001). However, in ventilated patients an initial $S_{cv}O_2$ below 70% remained significantly linked to day-28 over mortality by multivariate analysis (OR=2.36 [1.16-4.79]).

Role of hemoglobin concentration

The hemoglobin concentration (Hb) at the exact time (within one hour) of inclusion was known for 297 patients (82%). There was a weak but significant positive correlation between initial Hb and initial $S_{cv}O_2$ in these patients (r^2=0.03; p=0.002). The median initial Hb was 11 g/dL (IQR: 9.6-12.8). Patients with initial Hb below the median value had a higher prevalence of $S_{cv}O_2$ value below 70% than patients with Hb above or equal to median value (39% [60/152] *vs.* 21% [31/145], respectively; p<0.0001).

In multivariate analysis, initial $S_{cv}O_2$ below 70% was significantly linked to day-28 mortality in the subgroup of patients with Hb below 11 g/dL (OR=2.58 [1.14-5.83]) but not in patients with Hb above this value (OR=1.79 [0.53-6.04]).

Role of initial body temperature

We found no correlation between initial body temperature and initial $S_{cv}O_2$ value (r^2=0.003) and no relation between the changes in both parameters between H0 and H6 (r^2=0.0004). Although in multivariate analysis a high body temperature appeared to independently protect against day-28 death (OR=0.78 [0.62-0.98] for each 1-

Celsius degree increase in body temperature), an initial $S_{cv}O_2$ below 70% was significantly associated with day-28 mortality either when initial body temperature was below (OR=3.03; 95%CI: 1.07-8.55; p= 0.036) or above (OR=2.91; 95%CI: 1.00-8.44; p= 0.049) the median value of 37.2°C .
